# Blood microRNAs in Low or No Risk Ischemic Stroke Patients

**DOI:** 10.3390/ijms14012072

**Published:** 2013-01-22

**Authors:** Jun Rong Tan, Kay Sin Tan, Yu Xuan Koo, Fung Lin Yong, Chee Woon Wang, Arunmozhiarasi Armugam, Kandiah Jeyaseelan

**Affiliations:** 1 Department of Biochemistry and Neuroscience Research Centre, Centre for Translational Medicine, Yong Loo Lin School of Medicine, National University of Singapore, 8 Medical Drive, 117597, Singapore; E-Mails: a0030915@nus.edu.sg (J.R.T.); kooyuxuan@hotmail.com (Y.X.K.); bchaa@nus.edu.sg (A.A.); 2 Department of Medicine, Faculty of Medicine, University Malaya, Kuala Lumpur 50603, Malaysia; E-Mails: tanks@ummc.edu.my (K.S.T.); flyong88@yahoo.com (F.L.Y.); 3 Department of Biochemistry, Faculty of Medicine, MAHSA University College, 59100 Kuala Lumpur, Malaysia; E-Mail: wang.chee@mahsa.edu.my; 4 Department of Anatomy and Developmental Biology, School of Biomedical Sciences, Faculty of Medicine, Nursing and Health Sciences, Monash University, Clayton, Victoria 3800, Australia

**Keywords:** stroke, microRNA, etiology, modified Rankin Score

## Abstract

Ischemic stroke is a multi-factorial disease where some patients present themselves with little or no risk factors. Blood microRNA expression profiles are becoming useful in the diagnosis and prognosis of human diseases. We therefore investigated the blood microRNA profiles in young stroke patients who presented with minimal or absence of risk factors for stroke such as type 2 diabetes, dyslipidemia and hypertension. Blood microRNA profiles from these patients varied with stroke subtypes as well as different functional outcomes (based on modified Rankin Score). These microRNAs have been shown to target genes that are involved in stroke pathogenesis. The findings from our study suggest that molecular mechanisms in stroke pathogenesis involving low or no risk ischemic stroke patients could differ substantially from those with pre-existing risk factors.

## 1. Introduction

Ischemic stroke is a multi-factorial disease with multiple causes, and it constitutes one of the leading causes of adult disability worldwide [1]. The underlying cause of ischemic stroke is the occlusion of a cerebral artery leading to a lack of perfusion to the affected part of the brain [2,3]. There are multiple risk factors such as hypertension, atherosclerosis, type 2 diabetes, and smoking and alcohol consumption, which predispose individuals to greater risk of developing ischemic stroke [4]. Nevertheless, ischemic stroke is also becoming prevalent among young adults who do not exhibit any of the known risk factors.

Currently, the diagnosis for stroke is based on results from clinical examination, imaging and blood (protein) analyses. These diagnostic procedures are inadequate to understand the etiology of stroke in young adults who present themselves with no risk factors. In recent years, microRNAs (miRNAs) have been shown to be involved in the pathophysiology of various types of diseases including ischemic stroke [5–9]. MiRNAs are small, endogenous, non-coding RNAs that regulate gene expression in diverse biological processes [10,11]. Recently, circulating mRNA [12–14] and blood miRNA profiles [15–19] have been shown to provide vital clues as to the progression of cerebral ischemia. Most of these studies are carried out in *in vivo* animal models and *in vitro* conditions. Notably, most of the animal models for stroke present little or no pre-existing risk factors for stroke. Hence, the information about low risk ischemic stroke patients is thought to bridge the gap in the knowledge between experimental data and clinical data and to provide some insight into molecular mechanisms underlying ischemic stroke in young adults.

Previously, we had demonstrated that blood miRNAs display differential expression in young stroke patients of different stroke subtypes and functional outcomes [15]. These patients also presented with one or more risk factors. Increasing reports are being published to show the involvement of miRNAs in the pathology of type 2 diabetes, hypertension, the progression of atherosclerosis and detection of these as circulating miRNAs [20–23]. The differences in miRNA profiles in stroke patients presented with pre-existing risk factors are likely to be the result of the different co-morbidities as well. Therefore, the main aim of this study is to characterize the miRNA profiles from low/no risk young ischemic stroke patients and correlate them to cerebrovascular lesion caused by cerebral ischemia.

## 2. Results and Discussion

**Figure 1:**
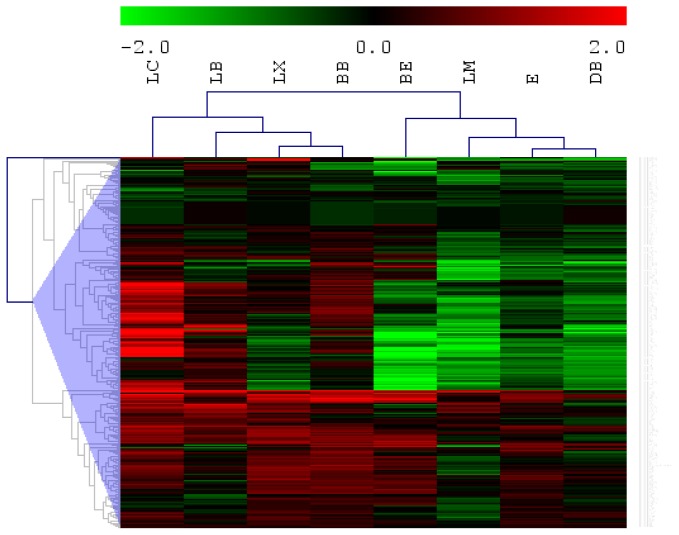
Hierarchical clustering of low/no risk ischemic stroke. Hierarchical clustering of blood miRNA profile of low/no-risk stroke patients (*n* = 8). Microarray data was normalized by average normalization using endogenous, small RNA controls on the microarray chip. For differential miRNA expression, the data was then normalized to the miRNA expression of the normal controls. The average intensities of each miRNA had been filtered by statistical testing (*t*-test, *p* < 0.05), normalized to the control readings and expressed as fold change and was selected for constructing the heat map. Green represents down-regulation while red represents up-regulation.

The results presented here were obtained from selected young ischemic stroke patients without pre-existing risk factors and represent unique miRNA profiles following ischemic stroke (Supplementary Data Table 1). A total of 293 miRNAs (*p* < 0.05) were detected in all the blood samples (Figure 1).

### 2.1. MicroRNAs That Show Common Expression in No Risk Ischemic Stroke

Twenty-one (21) miRNAs (hsa-miR-1258, -125a-5p, -1260, -1273, -149, -220b, -23a*, -25*, -26b*, -29b-1*, -302e, -34b, -483-5p, -488, -490-3p, -498, -506, -659, -890, -920, -934) were observed to have similar expression level in all ischemic stroke samples (BB, DB, E, LB, LC, LX, BE, LM; Table 1). Among them, miR-25*, -34b, -483-5p and miR-498 were found to be down-regulated in all cases. In our previous study on the young stroke patients with existing risk factors [15], we found only miR-25* to be expressed but it remained up-regulated. Thus, suggesting that these miRNAs could prove to be specific for stroke pathogenesis in low risk stroke patients, possibly presenting a different molecular mechanism for their stroke pathogenesis as compared to stroke in patients with pre-existing risk factors [15]. In order to relate these miRNA expression to their respective function in stroke pathogenesis, we analyzed the miRNA:mRNA target pair using the miRNA target prediction software, Targetscan (www.targetscan.org) [24,25]. We found 13 miRNAs (miR-1258, -125a-5p, -1260, -1273, -149, -220b, -302e, -34b, -490-3p, -506, -659, -920, -934) that showed similar expression in all ischemic stroke samples, target genes that are involved in proliferation, hemostasis, inflammation and oxidative stress processes (Supplementary Data Table 2). Stamova *et al.* [12] also reported that patients with ischemic stroke could be differentiated from healthy individuals based on a list of genes that are involved in inflammation and thrombosis. Notably, the up-regulated miR-1258 was demonstrated to target heparanase that has been speculated to be involved in astrogliosis [26,27], thus contributing to the pathology of ischemic stroke progression by encouraging the movement of reactive astrocytes to the infarct lesion. miR-506 had been demonstrated to target peroxisome proliferator-activated receptor alpha (PPAR-α) and administration of PPAR-α agonist suppresses the oxidative damage and inflammation during cerebral ischemia [28,29]. Since miR-506 was up-regulated in all ischemic stroke samples, this may be a cause for oxidative damage and inflammation during ischemic stroke. miR-659 had been shown to target a growth factor, progranulin (*GRN*; [30]), and the increased expression thereof may indicate the repression of *GRN*. In addition, it had been demonstrated that loss of function in GRN results in enhanced inflammation and neurodegeneration, which suggests that GRN may be involved in inflammation during cerebral ischemia [31]. Furthermore, increased expression of miR-125a-5p had been reported to activate p53 to promote apoptosis [32,33].

It is noteworthy that five miRNAs (hsa-miR-208a, -519d, -605, -634, -99b*) were found to be up-regulated only in large artery stroke samples (BB, DB, E, LB, LC, LX; Table 2). PPAR-α has been shown to be the target of the up-regulated miR-519d in large artery stroke (Supplementary Data Table 2; [34]). The activation of PPAR-α inhibits atherogenesis and stabilizes atherosclerotic plaque by reducing the expression of matrix metalloproteinase 9 [35]. Thus, this particular miRNA may have an expression pattern distinctive to large artery ischemic stroke that is caused by disruption of atherosclerotic lesions in the cerebral blood vessels. Among these miRNAs, only the expression of miR-634 correlated to our previous report on stroke with pre-existing risk factors [15].

### 2.2. Potential Subtype Differentiation Using miRNAs

Although the sample size for cardioembolic and lacunar stroke were insufficient to make any statistical validation, from this study using just one sample per category, we could postulate the usefulness of miRNAs in understanding the subtype specific molecular processes that differentiate large artery stroke from other subtypes of stroke. Further studies are needed with more samples in each category to confirm these observations. For example, hsa-miR-1246 and 513a-5p that are usually found up-regulated in all stroke subtypes with pre-existing risk factors [15] were found to be down regulated in cardioemblic stroke sample from a patient who had no apparent pre-existing risk factors (BE; Table 2). The miR-767-5p observed to be down-regulated in our cardioembolic stroke sample has also been reported to be down regulated in patients suffering from acute myocardial infarction [36]. Thus, expression of miR-767-5p may be specific to cardiac related ailments and could potentially show a unique expression pattern in patients with cardioembolic stroke. Lacunar stroke (again predicted from a single sample) on the other hand showed almost all miRNAs (Table 2) to be down-regulated except miR-377, -767-5p and 875-3p. The miR-377, -767-5p and -875-3p while being down-regulated in cardioembolic stroke (BE), were up-regulated in the large artery stroke samples. Lacunar stroke sample also showed miRNAs (miR-1274a, -1280, -200c*, -375, -494, -520d-5p, -551a, -629*, -656, -657, -664, -766; Table 2) to be down-regulated in contrast to large artery and cardioembolic strokes. With this preliminary information, we were still able to correlate the expression of these miRNAs to their respective target mRNAs as reported by Jickling *et al.* [13,14]. Based on our in silico analysis, the targets for these miRNAs were found to be involved in excitotoxicity, proliferation and inflammation processes (Supplementary Data Table 2). These observations are also consistent with the report on the differences in etiology between cardioembolic stroke and large artery stroke by Xu *et al.* [37]. Cardioembolic stroke was reported to be correlated to infection and increased inflammatory response [37] and has also been shown to exhibit higher inflammatory response as compared to large artery stroke.

### 2.3. MicroRNAs Associated with Functional Outcome in Large Artery Stroke

**Figure 2:**
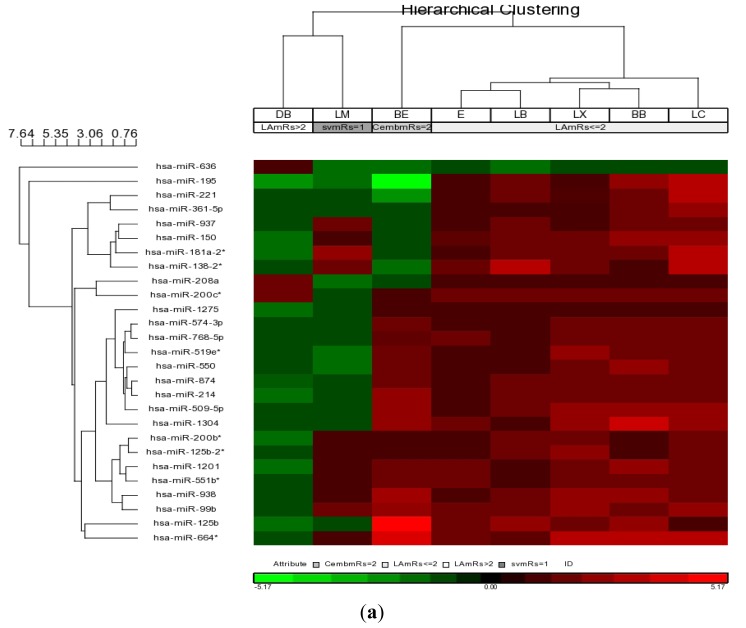

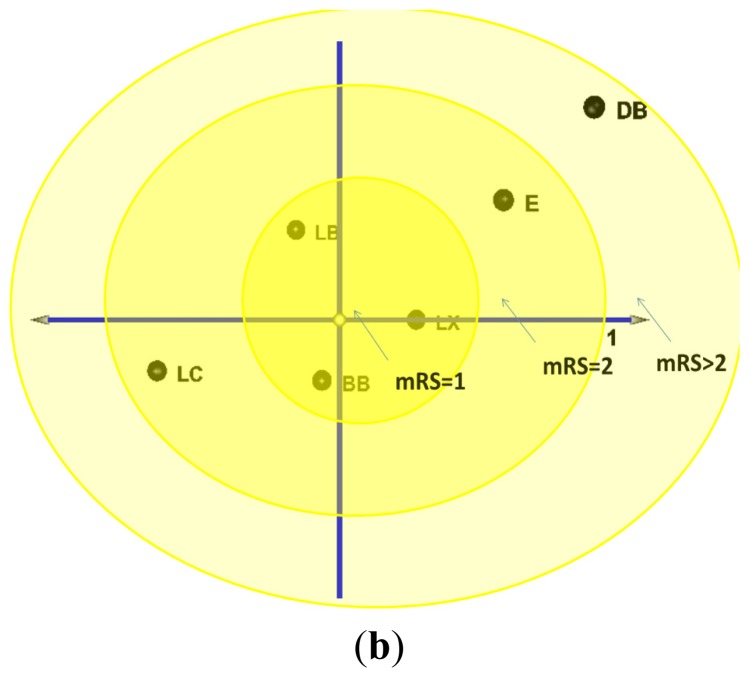
Hierarchical clustering and Principal component analysis (PCA) of low/no risk large artery ischemic stroke patients. (**A**) miRNA that were differentially expressed among the large artery stroke was analysed. 27 miRNA that showed significant correlation as corrected by FDR (*p-*value < 0.05) between large artery stroke with mRS ≤ 2 or mRS = 4. Hierarchical clustering was carried out on these miRNA for all the low/no-risk ischemic stroke patients (*n* = 8). Green rectangle represents down-regulation while red represents up-regulation of miRNA. (**B**) PCA of large artery ischemic stroke. A radial segregation of large artery stroke samples (BB, DB, E, LB, LC, LX) according to functional outcome (mRS) can be observed.

Hierachical clustering and Principal Component Analysis (PCA) analysis was performed on the blood miRNA profile of large artery ischemic stroke samples (BB, DB, E, LB, LC, LX; Figure 2A,B). Interestingly, the PCA profile showed a radial-like segregation of the samples according to functional outcome based on mRS. Large artery stroke samples with mRS = 1 (BB, LB, LX) clustered close together in the middle of the axis. The samples with mRS = 2 (E, LC) were located in a radial area adjacent to the first cluster, (Figure 2B) while a sample from a large artery stroke patient, DB, with mRS = 4, segregated furthest from the other large artery stroke samples (Figure 2B).

Based on the FDR correction and filtering *p*-value < 0.05 (Partek GS analysis), we found 27 hsa-miRNAs (miR-125b, -125b-2*, -1201, -1275, -1304, -138-2*, -150, -181a-2*, -195, -200b*, -200c*, -208a, -214, -221, -361-5p, -509-5p, -519e*, -550, -551b*, -574-3p, -636, -664*, -768-5p, -874, -937, -938, -99b) found to be differentially expressed between patients with good outcome (mRS ≤ 2; BB, LB, LC, LX and E) and patient with poor outcome (mRS = 4; DB: Figure 2A and Table 3). Of these miRNAs, only miR-208a and miR-636 exhibited opposite profile in DB (mRS = 4; Table 3) while the other 25 miRNAs were upregulated in the large artery stroke samples with mRS ≤ 2 (Table 3). Among them, miR-125b, -150, -214 and miR-221, have been reported to target genes that are relevant to the pathophysiology of ischemic stroke. miR-125b had been shown to target pro-apoptotic genes BCL-2 modifying factor (*BMF*) and *p53* [38,39]. This implies that there was higher expression of BMF and p53 in patient DB, which may account for increase in apoptotic process. Furthermore, miR-221 also targets another pro-apoptotic BCL2 binding component 3 (*BBC3/PUMA*), while miR-214 and miR-221 target the pro-apoptotic phosphatase and tensin homolog (*PTEN*) [40–42]. The effects of down-regulated miR-125b, -214 and -221 suggest an increase in apoptosis in DB and possibly account for the poor functional outcome. Moreover, endothelial cell migration that is implicated in angiogenesis is regulated by miR-150 [43]. Inhibition of angiogenesis could be the effect of the down-regulated miR-150. Hence, these cumulative effects of enhanced apoptosis and impairment of angiogenesis could have contributed to the poor functional outcome in DB (mRS = 4).

In an in-depth analysis of the clinical evaluation of the patients, DB and LB (both large artery stroke) showed that while both exhibited mRS = 4 upon admission, LB recovered to mRS = 1 but DB remained at mRS = 4, from hospitalization until discharge. The MRI and MRA images of DB (mRS = 4) and LB (mRS = 1) also presented no other confounding conditions that can affect the functional outcome of the patients (Supplementary Figure 1A–D). However, we found that the blood miRNA profiles reflected their respective stroke pathology. There were 167 hsa-miRNAs that showed opposite expression between DB (mRS = 4) and LB (mRS = 1; Supplementary Data Table 3), of which only 20 miRNAs were up-regulated and 147 miRNAs were down-regulated in DB (mRS = 4). Interestingly, some of these down-regulated miRNAs target pro-apoptotic and pro-inflammatory molecules. This shows that there could be an increased rate of cell death/apoptosis occurring in DB. Of these down-regulated miRNAs in DB, miR-126 and miR-146a targeting genes encoding vascular cell adhesion molecule 1 (*VCAM1*) and toll-like receptor 4 (*TLR4*) respectively can be associated with the functional outcome during ischemic stroke [44,45]. These correspond to previous reports [46,47] that increased expression of *VCAM1* and *TLR4* leads to poor outcome in stroke patients.

Therefore, we could conclude that the changes in miRNAs expression begin as a response to the injuries in the brain. However, as time progresses, these changes could lead to severity of stroke. Hence, we believe that modulating these differentially expressed miRNAs could prove useful in stroke therapy.

## 3. Experimental Section

### 3.1. Standard Protocol Approvals, Registrations, and Patient Consents

This study was approved by the Medical Ethics Committee of the University Malaya Medical Centre (UMMC), Kuala Lumpur, Malaysia and the Institutional Review Board (NUS-IRB Ref Code: 08-38; Approval: NUS-676) of the National University of Singapore (NUS). The original cohort of patients was derived from the UMMC Ischemic Stroke in Young Adults database kept since 2008 where over 200 patients have been collected. These patients were between the ages of 18 to 49 and were admitted via the Neurology service. The study protocol included a standard neurological evaluation with subsequent review and follow up as outpatients. Ischemic stroke was confirmed with either MRI or CT scan of brain. The patients’ functional outcome were reflected in the modified Rankin scale (mRS) where poor outcome is denoted by mRS > 2 and good outcome is denoted as mRS ≤ 2. Functional outcome was determined during admission and during blood collection. Demographic data, medical history and conventional vascular risk factors were abstracted from the medical records and entered into a standard computerized database.

In our database, risk factors were defined in the following manner [48]. Hypertension was defined as having blood pressure above 140/90 mmHg [48]. Dyslipidemic patients have a total cholesterol level ≥ 5.2 mmol/L, triglyceride level ≥ 1.8 mmol/L and HDL ≤ 1 mmol/L [48]. Diabetes mellitus was diagnosed as having a fasting blood glucose > 6.1 mmol/L or HbA1C ≥ 7% [48]. Smokers were defined as subjects who smoked 10 cigarettes per day for more than one year. Significant alcohol consumption was defined as taking 30 g of ethanol per day.

The ischemic patients were classified according to Trial of Org 10172 in Acute Stroke Treatment (TOAST) classification for their stroke subtypes, which are as follows: large-artery atherosclerosis, cardioembolic, small-vessel occlusion (lacunar) and undetermined etiology depending on presentation [49].

### 3.2. Blood Collection and Total RNA Isolation

Blood from stroke patients without any risk factor or minimal risk factor (*n* = 8) were collected (once from each patient) between 2 and 24 months from stroke onset. Fresh blood (5 mL) samples that were collected (using a syringe without any additives) from stroke patients (*n* = 8) and normal individuals (*n* = 4) were immediately added as 0.5 mL per sterile microfuge tubes containing 1.3 mL RNALater (Ambion, Austin, TX, USA). These samples were stored at −80 °C until further processing.

Total RNA (+miRNAs) was isolated from whole blood using the Ribopure™ Blood RNA isolation Kit (Ambion, Austin, TX, USA) according to manufacturer’s protocol. RNA concentration was determined using ND-1000 Spectrophotometer (Nanodrop™, Rockland, DE, Wilmington, DE, USA) and the integrity of RNA samples were verified using denaturing gel electrophoresis (15% polyacrylamide).

### 3.3 MicroRNA Microarray and Analysis

Five hundred nanograms (500 ng) of total RNA isolated from peripheral whole blood were used for hybridization. MicroRNAs were labeled using miRCURY LNA™ microRNA array Power Labeling kit (Exiqon, Vedbaek, Denmark) and hybridization was performed on the MAUI^®^ Hybridization system (Exiqon, Vedbaek, Denmark). The miRNA array (miRBase version 12) chips were scanned using Innoscan 700 scanner (Innopsys, France) with Mapix^®^ software (Innopsys, Carbonne, France). Microarray data was normalized by average normalization using endogenous, small RNA controls on the microarray chip. Basic statistical analysis was performed using Microsoft Excel (2010) data analysis such as ANOVA, student *t*-tests before selecting the differentially expressed miRNAs. Differential expression analysis of the miRNAs was performed using the FDR (Benjamini-Hochberg False Discovery Rate) correction (*p* < 0.05) as described in Partek^®^ Genomics Suite™ 6.6 Software (Partek Inc, St Louis, MI USA). Hierarchical clustering (HCL) and principal component analysis (PCA) were performed using TIGR MeV (TMeV) software and Partek^®^ Genomics Suite™ 6.6 Software (Partek Inc., St. Louis, MI, USA). *In silico* target prediction for miRNAs was conducted using Targetscan ([24,25]; www.targetscan.org).

## 4. Conclusions

In summary, we have described unique changes of circulating miRNA expression in young stroke patients with low/no risk factors. These miRNA profiles are likely to be specific to stroke pathogenesis and could improve the understanding of molecular mechanisms implicated in ischemic stroke and its prognosis. Based on *in silico* analysis, we found that specific clusters of miRNAs do correlate to the reported stroke pathology and these clusters differ from our previous report [15]. This suggests that the molecular basis of stroke pathology may be different in low/no risk patients as compared with patients with pre-existing risk factors [15]. Nevertheless, this study also supports our earlier findings that miRNA expression pattern could be used to identify stroke subtypes and functional outcomes. Although the sample size is rather low, the findings could be used as a proof of concept to highlight the importance further studies with larger cohort of stroke patients with low/no risk factors.

## Supplementary Information



## Figures and Tables

**Table 1:** MiRNAs (microRNAs) with similar expression patterns in all low-risk ischemic stroke samples. MiRNA expression is shown as fold change with respect to control samples.

hsa-miRNA	BB	DB	E	LB	LC	LX	BE	LM
miR-1258	2.33	1.57	1.94	1.87	4.90	3.35	2.35	1.56
miR-125a-5p	1.52	1.06	1.45	1.40	1.97	2.18	1.37	1.39
miR-1260	1.78	1.60	1.28	1.48	2.35	2.27	1.42	1.10
miR-1273	5.57	1.30	2.80	4.43	4.91	4.52	4.23	2.73
miR-149	1.75	1.67	1.79	1.43	2.40	1.59	1.08	1.15
miR-220b	2.93	1.81	1.99	1.92	2.95	3.08	2.49	1.27
miR-23a*	1.57	1.01	1.79	1.16	1.50	1.85	1.69	1.39
miR-25*	−1.57	−1.32	−1.14	−1.36	−1.71	−1.20	−1.17	−1.09
miR-26b*	2.80	1.61	2.04	1.39	3.08	2.30	2.50	1.09
miR-29b-1*	1.44	1.47	1.57	1.17	1.45	1.63	1.55	1.13
miR-302e	1.60	1.06	1.27	1.89	1.75	1.70	1.06	1.43
miR-34b	−1.28	−1.37	−1.18	−1.18	−1.26	−1.70	−1.20	−1.86
miR-483-5p	−1.10	−1.45	−1.23	−1.09	−1.66	−1.04	−1.07	−1.49
miR-488	1.41	1.07	1.28	2.23	1.82	2.32	1.20	1.69
miR-490-3p	4.04	1.65	2.13	1.05	2.22	3.21	3.47	1.05
miR-498	−1.23	−1.24	−1.23	−1.53	−1.40	−1.03	−1.07	−1.20
miR-506	1.40	1.55	2.09	2.49	2.86	2.35	1.01	1.35
miR-659	1.53	1.02	1.16	1.01	1.51	1.44	1.47	1.08
miR-890	2.09	1.03	1.30	1.20	2.08	2.28	1.43	1.35
miR-920	2.55	1.22	1.93	1.31	1.59	2.34	2.67	1.42
miR-934	1.99	1.02	1.26	1.03	1.23	1.98	1.72	1.12

**Table 2:** MiRNAs associated with stroke subtypes. MiRNA expression is shown as fold change with respect to control samples. The first 5 comprise of miRNAs with unique expression in large artery stroke compared to other subtypes.

hsa-miRNA	Large Artery						Cardioembolic	Lacunar
	
	BB	DB	E	LB	LC	LX	BE	LM
miR-208a	1.10	1.38	1.12	1.14	1.26	1.17	−1.23	−1.38
miR-519d	1.38	1.31	1.07	1.08	1.54	1.61	−1.09	−1.86
miR-605	1.71	1.15	1.34	1.11	1.75	2.11	−1.04	−1.79
miR-634	1.33	1.08	1.16	1.30	1.36	1.56	−1.09	−1.35
miR-99b*	1.17	1.17	1.22	1.17	1.27	1.16	−1.03	−1.07
miR-1246	−1.04	−1.23	−1.18	−1.06	−1.40	−1.20	1.03	−1.26
miR-377	2.76	1.07	1.14	3.59	4.51	1.61	−1.34	3.75
miR-513a-5p	−1.65	−1.29	−1.37	−1.30	−2.00	−1.24	1.15	−1.07
miR-767-5p	1.19	1.16	1.08	2.46	2.36	1.60	−1.10	1.84
miR-875-3p	1.75	1.05	1.19	2.36	1.92	1.94	−1.24	2.19
miR-1274a	1.19	1.42	1.26	1.09	1.89	1.43	1.04	−1.48
miR-1280	1.59	1.31	1.21	1.23	1.77	1.76	1.29	−1.46
miR-200c*	1.65	1.34	1.71	1.61	1.84	1.87	1.10	−1.02
miR-375	1.17	1.08	1.35	1.13	1.42	1.55	1.09	−1.33
miR-494	1.25	1.42	1.40	1.07	1.43	1.33	1.22	−1.09
miR-520d-5p	2.03	1.27	1.33	1.38	2.37	2.13	1.41	−1.16
miR-551a	1.32	1.15	1.18	1.30	1.26	1.11	1.05	−1.02
miR-629*	1.51	1.50	1.59	1.10	1.62	1.88	1.12	−1.37
miR-656	1.58	1.28	1.64	1.03	1.45	1.89	1.42	−1.25
miR-657	1.57	1.13	1.67	1.10	1.26	2.25	1.35	−1.05
miR-664	1.35	1.12	1.17	1.21	1.13	1.37	1.12	−1.02
miR-766	2.05	1.25	1.18	1.34	1.89	1.85	1.71	−1.32

**Table 3:** MiRNAs associated with functional outcome in large artery stroke samples only. Differential expression of miRNA (mRS < 2 *vs.* mRS > 2) was determined using FDR correction (*p-*value < 0.05). MiRNA expression is shown as fold change with respect to miRNA expressed in large artery (LA) stroke with mRS > 2.

hsa-miRNA	*p*-value (LA mRs ≤ 2 *vs.* LA mRs > 2)	Fold-Change (LA mRs ≤ 2 *vs.* LA mRs > 2)
hsa-miR-1201	0.0018	9.3616
hsa-miR-125b	0.0044	8.7958
hsa-miR-125b-2*	0.0039	6.4179
hsa-miR-1275	0.0000	6.5432
hsa-miR-1304	0.0258	9.3241
hsa-miR-138-2*	0.0213	8.2105
hsa-miR-150	0.0023	11.3980
hsa-miR-181a-2*	0.0106	11.4422
hsa-miR-195	0.0205	15.1663
hsa-miR-200b*	0.0004	6.4327
hsa-miR-200c*	0.0371	1.3122
hsa-miR-208a	0.0289	−1.1674
hsa-miR-214	0.0011	8.6163
hsa-miR-221	0.0363	8.0340
hsa-miR-361-5p	0.0029	6.3937
hsa-miR-509-5p	0.0018	9.0491
hsa-miR-519e*	0.0081	6.7593
hsa-miR-550	0.0053	7.0590
hsa-miR-551b*	0.0012	6.0334
hsa-miR-574-3p	0.0021	5.7031
hsa-miR-636	0.0001	−5.3117
hsa-miR-664*	0.0146	13.2992
hsa-miR-768-5p	0.0006	6.2516
hsa-miR-874	0.0017	7.7018
hsa-miR-937	0.0019	6.2523
hsa-miR-938	0.0029	7.4285
hsa-miR-99b	0.0048	7.5901
