# Supplementary material for: Blood microRNAs in Low or No Risk Ischemic Stroke Patients
Source: Int J Mol Sci. 2013 Jan 22;14(1):2072–84. doi: 10.3390/ijms14012072 (PMC3565366; doi:10.3390/ijms14012072)
Supplement: Supplementary file 1 [file ijms-14-02072-s001.pdf]

Supplementary Information

Figure S1. MRI and MRA scans of DB (mRS = 4) and LB (mRS = 1). (**A**) T2-weighted MRI image of DB (mRS = 4). The black arrow shows the ischemic lesion at the right basal ganglia; (**B**) T2-weighted MRI image of LB (mRS = 1). The black arrow shows the ischemic lesion at the left basal ganglia; (**C**) MRA image of DB (mRS = 4). The white arrow shows the region of vessel that is occluded; (**D**) MRA image of LB (mRS = 1). The white arrow shows irregularities in the vessel. The MRA image of DB (mRS = 4) showed that the ischaemia was due to the stenosis in the right MCA and resulted in an ischaemic lesion at the right basal ganglia indicated in MRI image (4A, 4C). In contrast, the MRA images of LB (mRS = 1) indicated that there are irregularities in the left ICA which could be the source of the occlusion and the MRI images depicted the ischaemic lesion at the left basal ganglia (4B, 4D).

4A

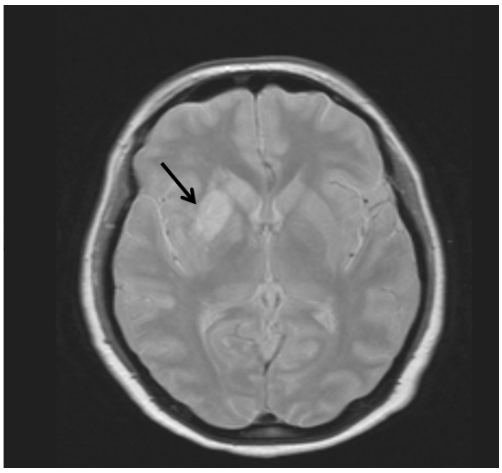

4B

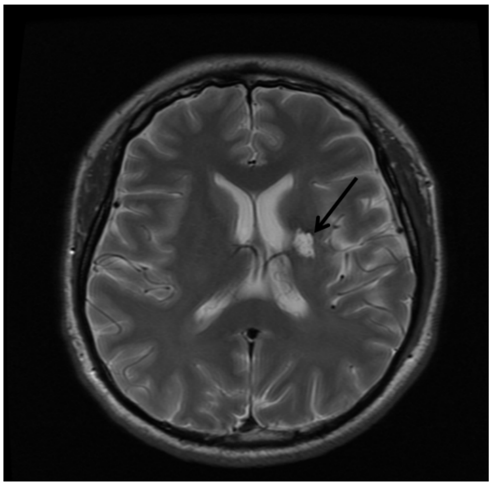

4C

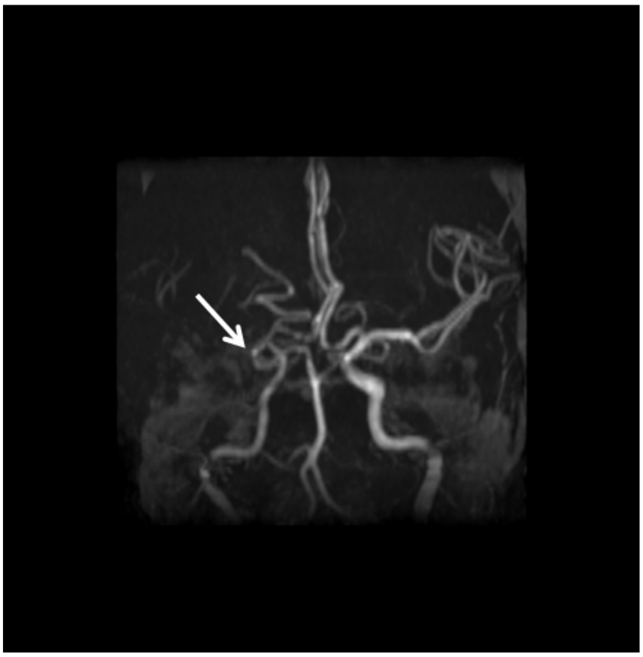

Figure S1. *Cont.*

4D

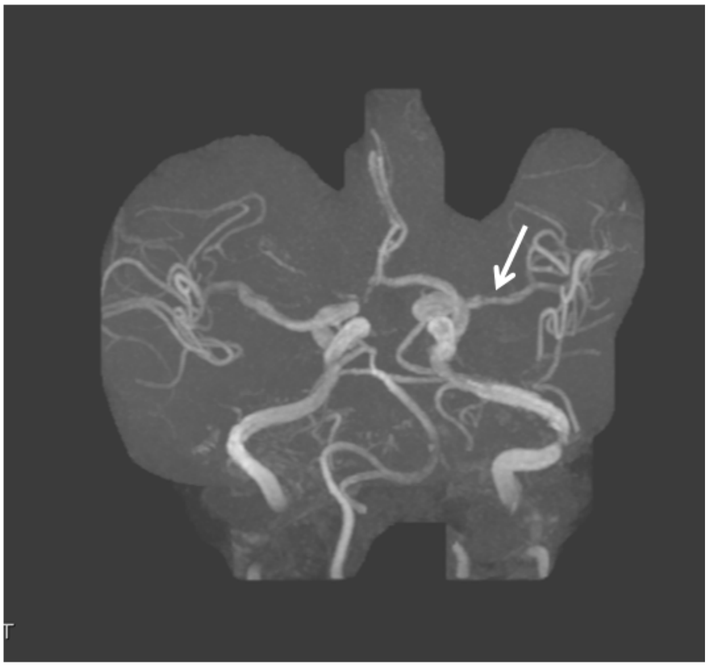

Table S1. Demographics of low/no risk stroke patients.

**Gender**	
Male, *n* (%)	6 (75%)
Female, *n* (%)	2 (25%)
Age (mean ± standard deviation)	36.7 ± 8.48
**Stroke Subtype**	
Large artery stroke, n	6 (BB, DB, E, LB, LC, LX)
Cardioembolic, n	1 (BE)
Small vessel, n	1 (LM)
**Functional outcome (Modified Rankin Scale)**	
mRS ≤ 2, *n*	7 (BB, BE, E, LB, LC, LM, LX)
mRS > 2, *n*	1 (DB)
**Blood Test**	
Cholestrol Level, mmol/L (mean)	5.2
Triglyceride Level, mmol/L (mean)	1.57
HDL, mmol/L (mean)	1.1
LDL, mmol/L (mean)	3.51
Fasting Glucose level, mmol/L (mean)	5.81
HbA1c, % (mean)	5.72

Table S2. MiRNAs and their predicted targets based on Targetscan. Some of the targets that have been validated are presented with respective reference in superscript, as underlined bold fonts.

MicroRNA	Gene	Stroke subtype
**miR-1258**	**chemokine-like factor; heparanase**[38]	**Ischemic stroke**
**miR-125a-5p**	Fatty acyl CoA reductase 2; ectonucleoside triphosphate diphosphohydrolase 1; RAS (RAD and GEM)-like GTP binding 2	
**miR-1260**	GRB2-associated binding protein 1	
**miR-1273**	RAS (RAD and GEM)-like GTP binding 2	
**miR-149**	antagonist of mitotic exit network 1 homolog (S. cerevisiae); leukotriene B4 receptor	
**miR-220b**	chromatin modifying protein 1B; thrombomodulin	
**miR-302e**	FYVE, RhoGEF and PH domain containing 4	
**miR-34b**	thrombomodulin	
**miR-490-3p**	bone marrow stromal cell antigen 1; FCH domain only 2	
**miR-506**	C-type lectin domain family 4-member E; cytoplasmic polyadenylation element binding protein 2; lysosomal-associated membrane protein 2; Quaking homolog; KH domain RNA binding (mouse); zinc finger protein 608; **peroxisome proliferator-activated receptor alpha** [42]	
**miR-659**	coagulation factor V (proaccelerin, labile factor); cytoplasmic polyadenylation element binding protein 2; RAS (RAD and GEM)-like GTP binding 2; RNA binding motif protein 25; **progranulin** [44]	
**miR-920**	C-type lectin domain family 4-member E; GRB2-associated binding protein 1; leukotriene B4 receptor	
**miR-934**	C-type lectin domain family 4- member E	
**miR-1246**	chromosome 19 open reading frame 28; early B-cell factor 1; major histocompatibility complex, class II, DO alpha	**Cardioembolic stroke**
**miR-377**	CDNA FLJ58216 complete cds, highly similar to Zinc finger protein 539; glutamate receptor- metabotropic 5; major histocompatibility complex, class II, DO alpha; zinc finger protein 185 (LIM domain)	
**miR-513a-5p**	Fc receptor-like 1; lipoma HMGIC fusion partner; major histocompatibility complex, class II, DO alpha; phosphoinositide-3-kinase, class 2, beta polypeptide; Rho guanine nucleotide exchange factor (GEF) 12; zinc finger protein 185 (LIM domain)	
**miR-767-5p**	adaptor-related protein complex 3, sigma 2 subunit; CD46 molecule, complement regulatory protein; churchill domain containing 1; ectonucleotide pyrophosphatase/phosphodiesterase 2; lipoma HMGIC fusion partner; major histocompatibility complex, class II, DO alpha; Rho guanine nucleotide exchange factor (GEF) 12;	
	Rho guanine nucleotide exchange factor (GEF) 5	
**miR-875-3p**	early B-cell factor 1; Fc receptor-like 1; lipoma HMGIC fusion partner; major histocompatibility complex, class II, DO alpha; phosphoinositide-3-kinase, class 2, beta polypeptide; protein tyrosine phosphatase, non-receptor type 20A /// protein tyrosine phosphatase, non-receptor type 20B; Rho guanine nucleotide exchange factor (GEF) 12	

Table S2. *Cont.*

**miR-1274a**	2'-5'-oligoadenylate synthetase-like; A kinase (PRKA) anchor protein (yotiao) 9; benzodiazapine receptor (peripheral) associated protein 1; chemokine (C-C motif) ligand 4; G protein-coupled receptor 56; Leucine-rich repeat-containing G protein-coupled receptor 6; Pyridoxal-dependent decarboxylase domain containing 1; quaking homolog, KH domain RNA binding (mouse); rhotekin 2; serine/threonine kinase 4; sperm associated antigen 17; synaptogyrin 1; syntaxin 7; TBC1 domain family, member 12; T-box 21; v-erb-b2 erythroblastic leukemia viral oncogene homolog 2, neuro/glioblastoma derived oncogene homolog (avian)	
**miR-1280**	2'-5'-oligoadenylate synthetase-like; benzodiazapine receptor (peripheral) associated protein 1; chemokine (C-C motif) ligand 3 /// chemokine (C-C motif) ligand 3-like 1 /// chemokine (C-C motif) ligand 3-like 3; KIAA1618; pleckstrin homology domain containing, family F (with FYVE domain) member 1; Runt-related transcription factor 3; synaptogyrin 1; syntaxin 16; TBC1 domain family, member 12; T-box 21; v-erb-b2 erythroblastic leukemia viral oncogene homolog 2, neuro/glioblastoma derived oncogene homolog (avian);	
**miR-375**	basonuclin 2; Choroideremia-like (Rab escort protein 2); quaking homolog, KH domain RNA binding (mouse); rhotekin 2; T-box 21; VAMP (vesicle-associated membrane protein)-associated protein A, 33kDa; v-erb-b2 erythroblastic leukemia viral oncogene homolog 2, neuro/glioblastoma derived oncogene homolog (avian)	
**miR-494**	basonuclin 2; benzodiazapine receptor (peripheral) associated protein 1; Grainyhead-like 2 (Drosophila); GRAM domain containing 3; guanylate binding protein 4; KIAA1618; leucine rich repeat containing 8 family, member B; Protease, serine, 23; Pyridoxal-dependent decarboxylase domain containing 1; rhotekin 2; serine/threonine kinase 4; sestrin 2; signal transducer and activator of transcription 1, 91kDa; solute carrier family 16, member 1 (monocarboxylic acid transporter 1); TBC1 domain family, member 12; tripartite motif-containing 4; Tubulin, epsilon 1; uveal autoantigen with coiled-coil domains and ankyrin repeats; VAMP (vesicle-associated membrane protein)-associated protein A, 33kDa	**Lacunar stroke**
**miR-520d-5p**	basonuclin 2; B-cell translocation gene 1, anti-proliferative; calmodulin 1 (phosphorylase kinase, delta); chemokine (C-C motif) ligand 2; chemokine (C-C motif) ligand 3 /// chemokine (C-C motif) ligand 3-like 1 /// chemokine (C-C motif) ligand 3-like 3; Choroideremia-like (Rab escort protein 2); family with sequence similarity 105, member A; G protein-coupled receptor 56; Grainyhead-like 2 (Drosophila); guanylate binding protein 4; NEDD4 binding protein 2; Protease, serine, 23; Pyridoxal-dependent decarboxylase domain containing 1; quaking homolog, KH domain RNA binding (mouse); rhotekin 2; Runt-related transcription factor 3; serine/threonine kinase 4; solute carrier family 16, member 1 (monocarboxylic acid transporter 1); TBC1 domain family, member 12; T-box 21; tripartite motif-containing 4; Tubulin, epsilon 1; VAMP (vesicle-associated membrane protein)-associated protein A, 33kDa; v-erb-b2 erythroblastic leukemia viral oncogene homolog 2, neuro/glioblastoma derived oncogene homolog (avian)	

Table S2. *Cont.*

**miR-551a**	basonuclin 2	
**miR-656**	basonuclin 2; carbohydrate (N-acetylglucosamine-6-O) sulfotransferase 2; Choroideremia-like (Rab escort protein 2); Grainyhead-like 2 (Drosophila); interleukin 8; NEDD4 binding protein 2; Protease, serine, 23; Pyridoxal-dependent decarboxylase domain containing 1; rhotekin 2; Runt-related transcription factor 3; signal transducer and activator of transcription 1, 91kDa; solute carrier family 16, member 1 (monocarboxylic acid transporter 1); synaptogyrin 1; syntaxin 16; TBC1 domain family, member 12; transforming growth factor, beta receptor III; VAMP (vesicle-associated membrane protein)-associated protein A, 33kDa	
**miR-657**	basonuclin 2; guanylate binding protein 4; pleckstrin homology domain containing, family F (with FYVE domain) member 1; Protease, serine, 23; quaking homolog, KH domain RNA binding (mouse); sestrin 2; v-erb-b2 erythroblastic leukemia viral oncogene homolog 2, neuro/glioblastoma derived oncogene homolog (avian); Zinc finger protein 827	
**miR-664**	basonuclin 2;calmodulin 1 (phosphorylase kinase, delta); chemokine (C-C motif) ligand 2; Choroideremia-like (Rab escort protein 2); Craniofacial development protein 1; general transcription factor IIH, polypeptide 2, 44kDa;interleukin 8; myelin protein zero-like 3; Protease, serine, 23; quaking homolog, KH domain RNA binding (mouse); serine/threonine kinase 4; SLAM family member 7; solute carrier family 16, member 1 (monocarboxylic acid transporter 1); syntaxin 16; TBC1 domain family, member 12; tripartite motif-containing 4; uveal autoantigen with coiled-coil domains and ankyrin repeats; VAMP (vesicle-associated membrane protein)-associated protein A, 33kDa	
**miR-766**	basonuclin 2; benzodiazapine receptor (peripheral) associated protein 1; calmodulin 1 (phosphorylase kinase, delta); family with sequence similarity 105, member A; G protein-coupled receptor 56; Grainyhead-like 2 (Drosophila); guanylate binding protein 4; KIAA1618; myelin protein zero-like 3; Runt-related transcription factor 3; serine/threonine kinase 4; sestrin 2; suppressor of cytokine signaling 1; synaptogyrin 1; T-box 21; VAMP (vesicle-associated membrane protein)-associated protein A, 33kDa; Zinc finger protein 827	
**miR-519d**	**peroxisome proliferator-activated receptor alpha** [25]	**Large Artery Stroke**

Table S3. MiRNAs that showed opposite expression pattern in large artery stroke between patient with good outcome (LB; mRS = 1) and poor outcome (DB; mRS = 4). MiRNA expressions are shown as fold change with respect to control samples.

hsa-miRNA	LB (mRS = 1)	DB (mRS = 4)	hsa-miRNA	LB (mRS = 1)	DB (mRS = 4)	hsa-miRNA	LB (mRS = 1)	DB (mRS = 4)	hsa-miRNA	LB (mRS = 1)	DB (mRS = 4)	hsa-miRNA	LB (mRS = 1)	DB (mRS = 4)
**let-7a**	1.08	−2.54	**miR-142-3p**	1.08	−1.07	**miR-20b***	−1.14	1.08	**miR-362-3p**	1.62	−5.06	**miR-630**	1.08	−1.03
**let-7b**	1.42	−1.83	**miR-142-5p**	1.08	−1.15	**miR-210**	−1.80	1.19	**miR-362-5p**	1.45	−1.90	**miR-636**	−1.37	1.27
**let-7b***	1.74	−1.53	**miR-144***	1.09	−2.69	**miR-214**	1.58	−1.49	**miR-363**	1.28	−2.21	**miR-637**	1.52	−1.75
**let-7c**	1.58	−1.95	**miR-145**	−1.28	2.01	**miR-215**	1.55	−1.63	**miR-365**	1.42	−1.05	**miR-645**	2.76	−1.21
**let-7d**	1.64	−2.30	**miR-146a**	1.92	−1.23	**miR-22***	1.23	−1.44	**miR-374a**	1.75	−2.15	**miR-647**	1.59	−1.31
**let-7d***	1.30	−1.43	**miR-146b-5p**	2.10	−1.57	**miR-220c**	1.04	−1.83	**miR-374b**	1.53	−2.17	**miR-652**	1.10	−1.84
**let-7g**	1.20	−2.19	**miR-148a**	1.51	−2.28	**miR-221**	1.39	−1.26	**miR-378**	1.17	−1.60	**miR-660**	1.68	-2.75
**let-7i**	2.38	−2.00	**miR-148b**	1.56	−2.05	**miR-222**	1.32	−1.53	**miR-381**	1.58	−1.36	**miR-664***	1.32	−1.30
**miR-101**	1.33	−2.02	**miR-149***	1.51	−1.98	**miR-23a**	2.11	−1.21	**miR-425**	1.03	−2.21	**miR-671-5p**	−1.05	1.07
**miR-103**	1.06	−2.09	**miR-150**	1.54	−1.74	**miR-23b**	1.89	−1.22	**miR-425***	1.11	−1.60	**miR-7**	1.45	−1.58
**miR-106a**	1.24	−2.31	**miR-151-3p**	1.15	−1.37	**miR-24**	1.34	−2.03	**miR-484**	1.05	−1.11	**miR-768-5p**	1.18	−1.15
**miR-106b***	1.28	−2.08	**miR-151-5p**	1.46	−1.74	**miiR-25**	1.78	−2.05	**miR-487b**	−1.13	1.30	**miR-874**	1.40	−1.32
**miR-107**	1.34	−2.47	**miR-15a**	1.08	−1.17	**miR-26a**	2.08	−1.89	**miR-489**	1.80	−1.24	**miR-886-3p**	1.54	−3.40
**miR-1184**	1.81	−1.77	**miR-15b***	1.17	−2.00	**miR-27a**	1.54	−1.74	**miR-491-3p**	−2.28	1.82	**miR-886-5p**	1.21	−2.35
**miR-1201**	1.01	−1.64	**miR-16-2***	3.99	−2.41	**miR-28-5p**	1.24	−1.88	**miR-493***	3.40	−1.69	**miR-93**	1.08	−1.45
**miR-1248**	-1.94	1.29	**miR-17**	1.25	−2.22	**miR-299-3p**	-1.55	1.08	**miR-500***	1.02	−2.46	**miR-93***	1.43	−1.31
**miR-125b**	2.03	−1.39	**miR-17***	1.28	−1.68	**miR-29a**	1.30	−1.97	**miR-502-3p**	1.28	−2.05	**miR-933**	−1.20	1.09
**miR-125b-2***	1.41	−1.23	**miR-181a-2***	1.86	−1.75	**miR-29b**	1.09	−1.92	**miR-502-5p**	1.60	−1.41	**miR-937**	1.65	−1.19
**miR-126**	1.31	−2.38	**miR-182**	4.32	−3.27	**miR-300**	1.65	−1.65	**miR-509-5p**	1.86	−1.30	**miR-938**	1.51	−1.09
**miR-126***	1.28	−2.52	**miR-1827**	1.46	−1.43	**miR-301a**	1.50	−2.26	**miR-510**	1.81	−1.44	**miR-939**	1.30	−1.97
**miR-1261**	1.34	−1.32	**miR-183**	2.51	−2.50	**miR-30b**	1.10	−2.10	**miR-519e***	1.15	−1.09	**miR-96**	2.73	−2.69
**miR-1264**	−1.01	1.18	**miR-183***	1.12	−1.66	**miR-30c**	1.24	−2.27	**miR-525-5p**	-1.16	1.21	**miR-98**	1.33	−2.34
**miR-1265**	−1.24	1.13	**miR-185**	1.07	−1.08	**miR-30d**	1.33	−1.85	**miR-532-5p**	1.33	−2.51	**miR-99b**	1.77	−1.04
**miR-1270**	−1.12	1.83	**miR-185***	1.59	−1.59	**miR-30e**	1.32	−1.72	**miR-550**	1.10	−1.25			
**miR-1275**	1.25	−1.55	**miR-186**	1.27	−1.01	**miR-30e***	1.76	−2.55	**miR-551b***	1.11	−1.08			
**miR-1284**	1.05	−1.23	**miR-187***	1.29	−1.27	**miR-32**	1.73	−2.04	**miR-557**	1.48	−1.33			

Table S3. *Cont.*

**miR-1285**	1.06	−1.20	**miR-18a**	1.22	−1.68	**miR-32***	1.11	−1.16	**miR-574-3p**	1.20	−1.06
**miR-129***	−1.64	1.04	**miR-18b**	1.12	−1.72	**miR-324-5p**	1.18	−1.89	**miR-574-5p**	1.09	−1.12
**miR-129-5p**	1.33	−1.34	**miR-191**	1.07	−1.33	**miR-326**	1.12	−1.26	**miR-576-5p**	1.48	−1.01
**miR-1297**	1.76	−2.00	**miR-192**	1.89	−2.06	**miR-328**	−1.40	1.18	**miR-583**	1.11	−1.14
**miR-1304**	1.22	−1.08	**miR-193a-3p**	1.39	−1.35	**miR-331-5p**	1.02	−1.60	**miR-584**	−1.12	1.23
**miR-130a**	1.75	−1.98	**miR-193b***	1.03	−1.48	**miR-335**	2.89	−1.94	**miR-600**	−1.69	1.25
**miR-130b**	1.63	−2.35	**miR-195**	1.84	−2.02	**miR-340**	1.42	−1.23	**miR-601**	−1.51	1.25
**miR-138-2***	2.85	−1.02	**miR-200b***	1.52	−1.36	**miR-345**	1.16	−1.18	**miR-602**	1.22	−1.29
**miR-140-3p**	1.30	−1.62	**miR-20a***	2.01	−2.67	**miR-361-3p**	1.08	−1.68	**miR-625***	−1.07	1.10
**miR-140-5p**	1.19	−1.95	**miR-20b**	1.16	−2.18	**miR-361-5p**	1.14	−1.19	**miR-628-3p**	1.33	−1.88

© 2013 by the authors; licensee MDPI, Basel, Switzerland. This article is an open access article distributed under the terms and conditions of the Creative Commons Attribution license (http://creativecommons.org/licenses/by/3.0/).
